# From consumption to context: assessing poverty and inequality across diverse socio-ecological systems in Ghana

**DOI:** 10.1088/2515-7620/ad76ff

**Published:** 2024-09-17

**Authors:** Alicia C Cavanaugh, Honor R Bixby, Saeesh Mangwani, Samuel Agyei-Mensah, Cynthia Azochiman Awuni, Jill C Baumgartner, George Owusu, Brian E Robinson

**Affiliations:** 1 Department of Geography, McGill University, Montreal, Canada; 2 Institute of Public Health and Wellbeing, University of Essex, Colchester, United Kingdom; 3 IMBERSea, University of Ghent, Ghent, Belgium; 4 Department of Geography and Resource Development, University of Ghana, Accra, Ghana; 5 Department of Ethics, Equity and Policy, McGill University, Montreal, Canada; 6 Department of Epidemiology, Biostatistics & Occupational Health, McGill University, Montreal, Canada; 7 Institute of Statistical, Social & Economic Research (ISSER), University of Ghana, Accra, Ghana; 8 Centre for Migration Studies, University of Ghana, Accra, Ghana

**Keywords:** social-ecological systems, multiple dimensions of poverty, multiple dimensions of inequality, sub-Saharan Africa

## Abstract

Local social and ecological contexts influence the experience of poverty and inequality in a number of ways that include shaping livelihood opportunities and determining the available infrastructure, services and environmental resources, as well as people’s capacity to use them. The metrics used to define poverty and inequality function to guide local and international development policy but how these interact with the local ecological contexts is not well explored. We use a social-ecological systems (SES) lens to empirically examine how context relates to various measures of human well-being at a national scale in Ghana. Using a novel dataset constructed from the 100% Ghanian Census, we examine poverty and inequality at a fine population level across and within multiple dimensions of well-being. First, we describe how well-being varies within different Ghanian SES contexts. Second, we ask whether monetary consumption acts a good indicator for well-being across these contexts. Third, we examine measures of inequality in various metrics across SES types. We find consumption distributions differ across SES types and are markedly distinct from regional distributions based on political boundaries. Rates of improved well-being are positively correlated with consumption levels in all SES types, but correlations are weaker in less-developed contexts like, rangelands and wildlands. Finally, while consumption inequality is quite consistent across SES types, inequality in other measures of living standards (housing, water, sanitation, etc) increases dramatically in SES types as population density and infrastructural development decreases. We advocate that SES types should be recognized as distinct contexts in which actions to mitigate poverty and inequality should better incorporate the challenges unique to each.

## 1. Introduction

The experience of poverty and inequality are relative. How it feels to be poor depends on how different you are from the people around you and your ability to fully participate in society [1, 2]. In many cases, how one participates in society must be understood within local socio-cultural and ecological contexts [3]. Well-being—which we refer to as quality of life and the ability to meaningfully participate in society—depends on local infrastructure, economic context, climatic conditions, culture, and ways in which populations access and use environmental resources [4, 5]. Absolute poverty lines identify the level of income needed to achieve basic needs across all societies [6]. While the World Bank’s absolute poverty line of $2.15 a day aids in international comparison, it is less useful for understanding poverty in a particular place, as what constitutes minimum standards of living and nutritional needs can be highly location-specific [7]. Context affects the types and diversity of livelihood opportunities available, as well as the ability or need to use local resources [8, 9]. A social-ecological systems (SES) lens can help us understand and mitigate poverty and inequality, especially as the need for climate adaptation grows [10–13]. Thus, we use relative poverty to understand how it feels to be poor and how living standards differ in various SES contexts.

Consider, for example, the livelihood options and constraints of a financial consultant in Dakar, Senegal versus a subsistence groundnut farmer in eastern Mali that is only weakly integrated into the economic market system [14]. These two livelihood contexts have different opportunities and constraints, in large part due to interactions and dependencies in local environments. Yet we typically assess well-being in these two cases in the same way, namely through measures of consumption or income [15]. While monetary-based metrics enable cross-context comparisons, they provide little insight into different mechanisms for addressing poverty or livelihood issues at the local level.

Metrics used to define poverty and inequality are foundational for development policy. How these interact with the local ecological contexts are not well explored yet are critical for understanding local human well-being and strategies for community support [16]. For example, in some rural contexts, such as pastoral systems, livestock are often considered a better proxy for wealth compared to income or consumption [17–19]. Complications with consumption-based metrics have given rise to widespread adoption of multidimensional and capability approaches to measuring poverty, inequality, and well-being [20–30]. While multidimensional and locally-relevant indictors are being recognized [4, 11], there are no empirical and systematic examinations of how diverse SES context relate to human well-being, especially at a national scale.

In this paper, we use a multidimensional poverty and inequality approach to evaluate the appropriateness of consumption (monetary expenditures on goods and services used by households) as a proxy for human well-being within SES contexts in Ghana. To do so, we use a novel and unprecedentedly detailed dataset to explore differences in how a variety of well-being metrics relate to consumption among five anthropogenic biomes (‘anthromes’) [31]. Anthromes are a land system classification that incorporate the ecological context of anthropogenic systems [32] and provide a resource-based grounding for considering how poverty and inequality manifest in a particular location. This paper contributes to the literature in three ways. We (i) provide granular evidence of the distribution of demographic and housing indicators related to quality of life, (ii) provide evidence that populations are different (and should be considered differently) in various SES contexts, and (iii) show how SES context can guide policy priorities for poverty and inequality reduction.

## 2. Ghanaian context

Ghana has one of the fastest growing economies in Sub Saharan Africa [33, 34] yet in 2016 over 23% of the population still remained below the World Bank defined poverty line [35]. While there is a steady reduction in national poverty it is often a more rural phenomenon and is highly concentrated in the North [36]. Inequality has also steadily increased [36] as the benefits of growth have favored certain places over others [37]. Northern Ghana has seen the greatest rates of poverty reduction over the past two decades, though development in the rural north still lags behind the more urbanized southern regions [36, 38, 39]. Inequalities are increasing nationally due to disparities within districts and regions, rather than inequality between these areas [36, 38, 40].

Current patterns of poverty and inequality in Ghana have colonial roots related to investments directed toward resource-rich regions that had goods for the export market, ideal climates for cash crops (e.g. cocoa, coffee, rubber), and access to coastal trade [41]. Investments in infrastructure, hydroelectric projects, services, and housing were concentrated in the south [42]. Southern regions were connected by modernized transportation networks to increase agricultural and mineral exports [41] which spurred growth in the Accra, Kumasi, and Sekondi-Takoradi triangle [43]. Formerly a 19th century trading and food production center, economic activity in Northern Ghana slowed as a result of Southern bias in colonial expenditure [44, 45]. Northern Ghana’s arid climate and distance from the coast meant it received less investment, instead becoming an effective labor reserve for colonial production in the south [44]. However, the northern cities of Tamale, Wa, and Bolgatanga have experienced more recent steady growth [46, 47].

Ghana is also ecologically diverse, with distinct agroecological zones characterized by variations in climate, vegetation, and soil types, resulting in a wide variety of habitats and ecosystems [48]. Northern Ghana is relatively dry and characterized by savannah made up of open grasslands, scattered trees, and shrubs. Guinean savannah and a transitional zone occupy the middle part of the country, characterized by greater density of wooded areas and a diversity of plant and animal species. The transitional zone is a mix of forest, savannah, and grasslands habitats, lying between the savannah and more forested ecosystems to the south. The coastal regions of Ghana feature the coastal savannah to the east, including Accra, and coastal forest to the west. Southern inland Ghana around the Volta Basin is home to moist semi-deciduous forest [49]. This combination of dynamic and changing economic conditions across a range of ecological contexts makes Ghana an excellent candidate for exploring dynamics between well-being and SES contexts.

## 3. Data and methods

To assess how living standards vary across SES contexts, we pool multiple data sources available for Ghana. A challenge is synthesizing SES and measures of well-being at the same spatial scale. Below we describe methods for classifying SES types across Ghana, estimating consumption in small geographic units, defining measures of living standards (as proxies for household well-being), and assessing inequality.

### 3.1. Developing social-ecological system types

While some literature examining poverty and inequality has examined urban and rural differences [36–41], deeper examination of ecological context has been rare. Yet, especially from a development perspective, ‘rural’ is far too coarse to meaningfully capture characteristics of a social-ecological context to provide concrete insight for how living conditions or livelihood dynamics interact with poverty and inequality [50]. Agricultural households are quite market-oriented and integrated, while residents in largely undeveloped ‘wild’ lands may depend on local environmental resources for most of their daily needs. Poverty may be better understood when considering the systematically different resource and livelihood realities of people in, say, urban versus agricultural versus pastoral versus undeveloped contexts.

Here we use the major categories developed by the Anthromes project [31] to classify different social-ecological system (SES) types. While there is still a great deal of heterogeneity in these classes, it gives us a better sense of the ecological context in which a community dwells. The major anthrome classes [31] that exist in Ghana are: urban areas, mixed settlements & villages, croplands, rangelands, and wildlands. Urban can comprise business districts, slums, planned residential neighborhoods, and peri-urban areas. Villages and settlements (hereafter referred to as just settlements) are areas with mid-levels of population density and are a mix of more developed and rural environments which can also include towns, hamlets, and denser agricultural settlements. Croplands are areas with annual crops mixed with other land uses and land covers. Rangelands are areas dominated by livestock grazing, with few crops and forests. Wildlands are places with very limited development and low population densities but can represent areas with national parks as well as subsistence communities that rely on forest products.

The Anthromes database provides a global gridded classification at 5-arc minute resolution, resulting in some anthrome heterogeneity within districts. To account for variation between urban and rural contexts, we (a) masked out built-up areas [51] and (b) assigned each district a single population-weighted [51] anthrome (SES) class. EAs outside of urban areas were assigned the SES where the most people live in their district. We describe these two steps below.

#### 3.1.1. Identifying built-up areas

**Figure 1:**
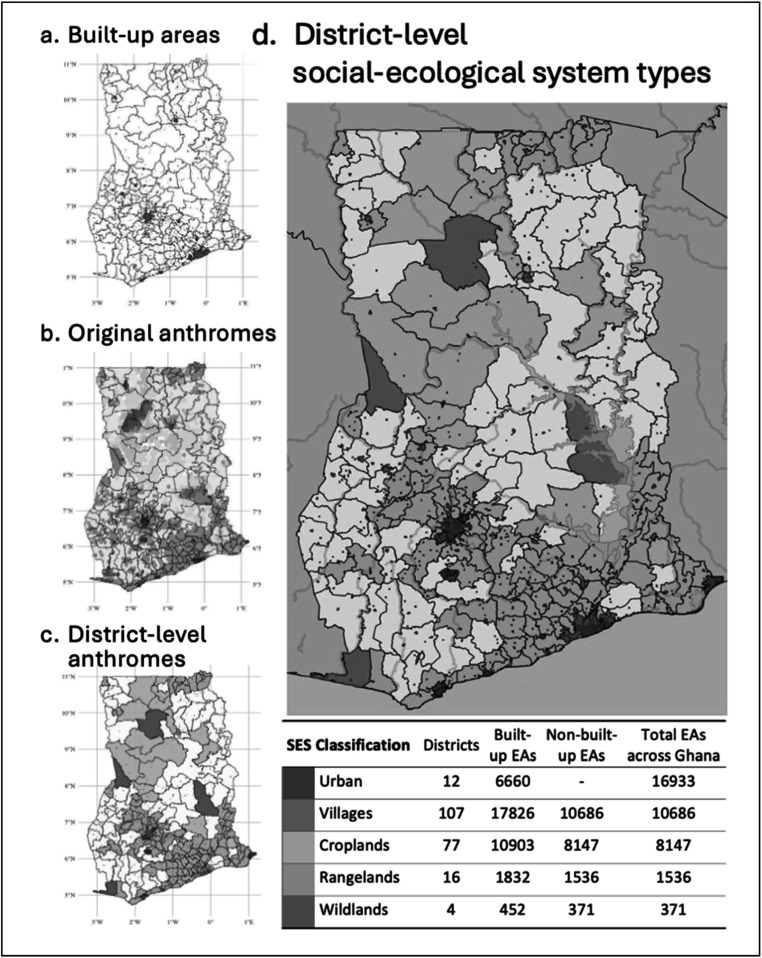
Classifying Social-Environmental System types. We (a) identify built-up areas, (b) overlay original anthrome land use classifications, and (c) assign districts a non-urban population-weighted anthromes type, resulting in (d) final resulting district-level social-ecological system types.

To identify the spatial extent of urban areas, we used the ESA Worldcover dataset [51]. ‘Built-up area’ indicates the spatial extent of urban infrastructure at a 10 m resolution. We used modal aggregation to and aggregate urban pixels at a 250 m^2^ resolution and identify large built-up areas in Ghana. The result was smoothed and polygonized to create contiguous units (figure 1(a)). Identified built-up areas aligned well with urban areas in the anthromes dataset, but also included smaller areas identified in the Worldcover data.

#### 3.1.2. Identifying district-level SES types using population-weights

Our Ghanian census data is comprised of 10 regions subdivided into 216 districts and 36,593 enumeration areas (EAs)—the smallest census administrative units in Ghana with an average population of ∼650 people. Shapefiles are only available for districts, but EAs are always labeled as rural or urban. We classified districts containing solely urban EAs (as identified in the Census) as ‘urban’ districts. For all other districts, we assigned an SES type based on the environment where most people live. We applied gridded population data (from the GPWv4 dataset [52]) to the masked anthrome data and assigned districts SES types based on the most common population-weighted anthrome pixels outside of urban areas in that district. In these districts, census-identified EAs were considered urban, and EAs identified as rural were assigned the district SES type. Figure 1 summarizes the steps taken to assign households in Ghana to one of five SES types and the number of districts and EAs in each category.

### 3.2. Developing consumption estimates for all households in Ghana

Since consumption data are not available in the census, we estimated consumption for 100% of households enumerated in the 2010 Ghanian Population and Housing Census [53], in collaboration with the Ghana Statistical Service (GSS), using small area estimation (SAE) methods ubiquitous in the poverty mapping literature [54, 55]. SAE borrows strength from a detailed, but less representative dataset to then predict an outcome for a representative but less detailed dataset. In our context, we first estimate predictors of equivalized consumption (log consumption in Ghana cedis (GH 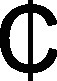 ) divided by the square root of the household size) using the 6th Ghana Living Standards Survey [56] in a linear mixed model that includes district-level random intercepts (SI table 1). Independent variables were selected from household characteristics measured in both the GLSS6 survey and the census using a LASSO regression model that assessed the top 20–25 most relevant correlates with GLSS consumption (SI table 1). Following procedures established at the GSS to account for regional-level heterogeneities, we estimated separate models for each of the 10 regions [57]. Then we applied the parameters and district-level random intercepts to predict consumption for Census households, and summarized poverty and inequality metrics at the EA level. Cavanaugh *et al* [58] provide more details on these procedures, but our dataset for analysis has EAs as the units of observation, with consumption and well-being metrics summarized at that level. We present results on relative poverty using consumption deciles based on the nationally-defined and regionally-defined distribution of consumption for two reasons. First, absolute poverty lines are set based on the ability to meet food and non-food needs at a national scale, creating a tendency to underestimate urban poverty [59–61]. Second, since Ghana’s absolute levels of poverty have recently risen, we want to understand the ability of those at the bottom to meet their basic needs relative to their neighbors. Deciles based on the national level show the inequalities throughout the country, while regionally-based deciles help account for variable living standards and aspects of relative poverty across regions.

While a sample of the latest Ghanian 2021 census was recently released, our current approach utilizes the full 100% sample of the 2010 census and the spatial distribution of the data, neither of which are available for analysis for the 2021 Census. The consumption estimates used in this study have also been used to examine spatial inequalities in air pollution [62], noise levels [63], child mortality [64], and drinking water [65] for the city of Accra.

### 3.3. Developing households well-being metrics

Well-being is a complex construct for which we do not have a singular ‘gold-standard’ measure [66, 67]. Accordingly, we use multiple indices that relate to well-being. Using data from the 100% Ghanian Census data, we chose variables that relate to education/access to information and household living standards in order to characterize two of the three dimensions of the Multidimensional Poverty Index [22] and the Human Development Index [68], as shown in table 1. We use the household head’s education attainment, mobile phone ownership, and internet access as measures of education and access to information as they can have a positive effect on the use of health services [69]. While this may be a relatively high standard as an average for the whole population, it is a reasonable comparative measure for the head of the household. Water sources, lighting, sanitation, and fuel sources are related to defense mechanisms that protect habitants from pollutants, disease, and environmental and social risks [70]. We categorize each household as having ‘improved’ versus ‘unimproved’ levels of these variables following UN Sustainable Development Goal guidelines and WHO/UNICEF’s Joint Monitoring Programme nomenclature [71–73].

**Table 1.** Household improved/unimproved living standards definitions.

	Improved	Unimproved
***Education & access to information***		
Education	Obtained high school degree	Not graduated high school
Mobile Phone	Owns a mobile phone	Does not own a mobile phone
Internet Access	Accesses the internet	Does not access the internet
***Living standards***		
Water	Pipe-borne inside dwelling	Dugout/Pond/Lake/Dam/Canal
	Pipe-borne outside dwelling	Other
	Bottled water	Rainwater
	Sachet water	River/Stream
	Bore-hole/Pump/Tube well	Tanker supply/Vendor provided
	Protected spring	Unprotected spring
	Protected well	Unprotected well
	Public tap/Standpipe	
Lighting	Electricity (mains)	Candle
	Electricity (private generator)	Crop residue
	Solar energy	Firewood
		Flashlight/Torch
		Gas lamp
		Kerosene lamp
		Other
Sanitation	KVIP	Bucket/Pan
	Pit latrine	No facilities (bush/beach/field)
	W.C.	Other
		Public toilet (WC, KVIP, Pit, Pan, etc)
Fuel	Electricity	Animal waste
	Gas	Charcoal
		Crop residue
		Kerosene
		None, no cooking
		Other
		Saw dust
		Wood

### 3.4. Assessing consumption versus well-being measures

We assess how ‘appropriate’ consumption appears to be as a measure of well-being and inequality, in terms of its agreement with other metrics within distinct SES classes. We judge this appropriateness of consumption in two ways. First, we examine the pair-wise joint distribution of our well-being indicators by deciles of consumption using a multidimensional inequality approach that captures the differences between distributions but also the correlations between well-being and consumption [26, 27]. To compare to other well-being measures, deciles of consumption are defined based on regional distributions to reflect potential local differences in consumption. Each household is assigned to a decile group based on their position in the distribution, with the first decile representing the 10% of households with the lowest levels of consumption, and 10th decile representing the greatest. A common relative poverty line is defined as 60% of the median consumption, which here aligns well with the 30th percentile threshold [74, 75]. Importantly, none of the well-being measures (section 3.3) were used as predictors of consumption in the SAE estimation procedure (section 3.2). Thus, the well-being measures and our estimates of consumption are statistically independent and valid for comparison [74]. While even broad overlap in variables (e.g., a well-being measure constructed in part from a variable included in estimation) could result in statistical circularity, all our well-being measures are broad aggregates of the percent of households in an EA with an ‘improved’ measure, thus is unlikely to be related to any of the ‘raw’ data used in the initial SAE procedure.

Second, we examine inequalities in consumption and other measures of well-being by calculating Gini coefficients to compare EA-level distributions for each indicator within each SES type [27, 29, 30]. Gini values range from perfect equality (zero) to perfect inequality (one). Gini coefficients are generally categorized as: < 0.3 is low inequality, 0.31–0.40 is medium inequality, 0.41–0.50 is high inequality, and > 0.5 is extremely high [75–77]. Our Gini coefficients are based on the percent (%) of households within an EA that have ‘improved’ measures (table 1) or, for consumption, EA-level average consumption measured in GH 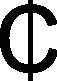 . Statistical offices do not generally report Gini values on EA-level summary statistics, but this gives us a common comparative metric by which to evaluation inequalities within SES types.

Since *consumption* is measured as continuous in Ghana cedis (GH 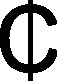 ), we can apply additional procedures to estimate the distribution of household-level consumption with each SES type to better allow for comparison with commonly reported estimates of poverty and inequality by national statistics offices. To estimate household-level inequality, however, we first note that Gini coefficients are sensitive to the skewness of underlying distribution of the data. Our raw modeled consumption estimates do not capture long tails in the true empirical distribution (predictions from models do not predict outliers or long tails of distributions by design). To allow our data to match the true empirical distribution of income, we applied ‘mean-constrained integration over brackets’ methods [78] to our estimates of EA-level average consumption. This effectively proportionally rescales our modeled estimates to match the range, variance, and non-parametric shape of the true empirical distribution as measured in the GLSS6. We then use this estimated household distribution to estimate a household-level Gini that represents consumption inequality.

## 4. Results

### 4.1. Consumption distributions differ across SES types

**Figure 2.**
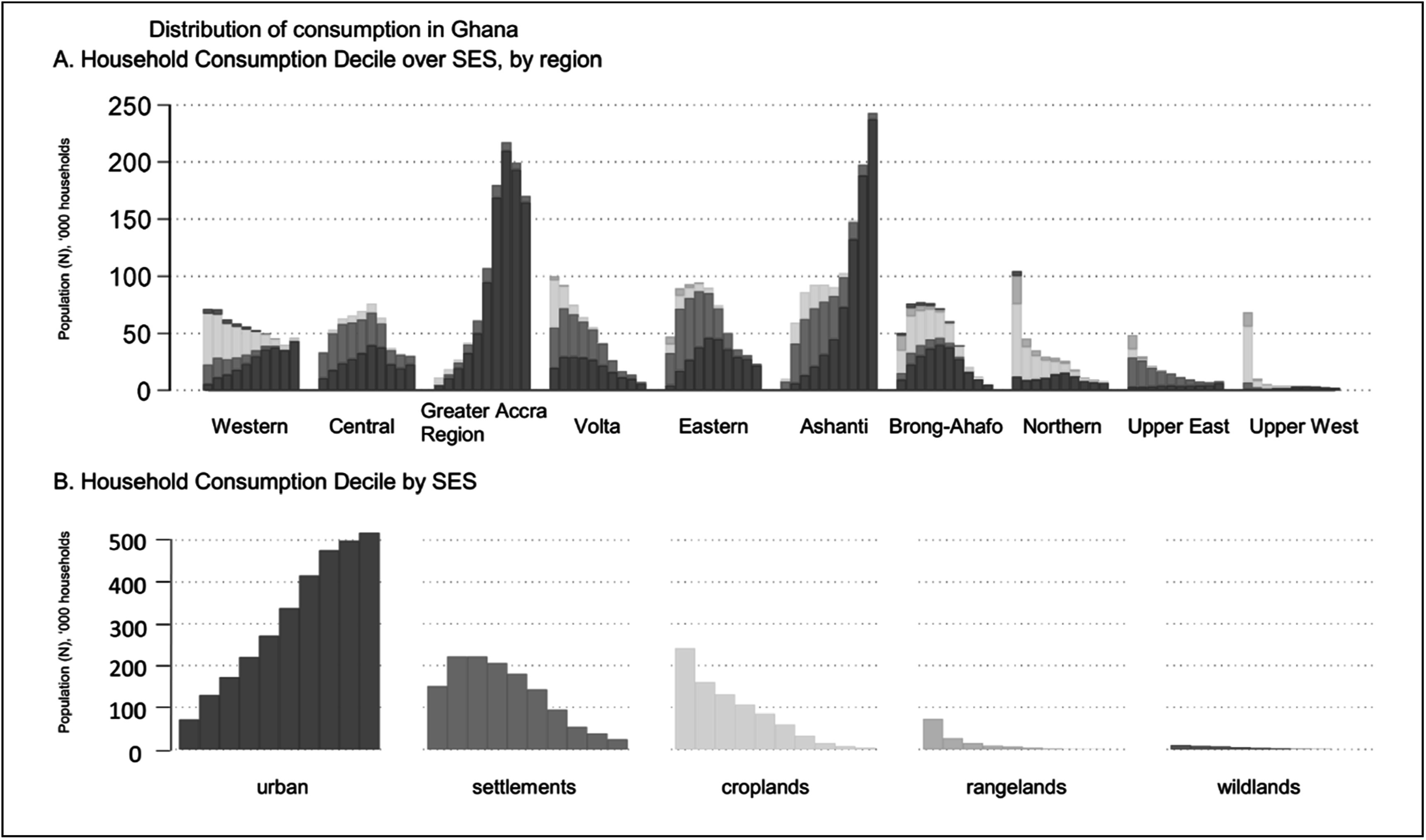
National distribution of consumption across regions and SES types. Count of households in each consumption group (defined by nationally-determined deciles) by administrative regions (A) and socio-environmental systems (B). National consumption deciles, ordered from left to right, from poorest to most affluent.

Figure 2 shows how the national distribution of consumption varies across regions (figure 2(a)), which is the way we might typically and intuitively think of poverty and inequality in a country. Rural and poor areas have low levels of consumption that skew toward lower consumption deciles, such as the regions of Northern, Upper East, and Upper West. These distributions are colored by the SES types represented within, and we see those regions have far fewer urban inhabitants with much greater representation of croplands (yellow), rangelands (light green), and settlements (orange).

The distribution of consumption by SES type (figure 2(b)) reveals very different patterns. Over SES types, the positive (urban) and negative (settlements, croplands, rangelands, and wildlands) relationships between population numbers and consumption are striking. Despite significant variability in living environments within settlements and urban systems, they have the highest number of households in the lower 30th percentile of the consumption distribution, i.e., living in relative poverty (585,132 and 524,140, respectively; figure 2(b)). While the poor make up 44% of households in the settlements category, only 17% are poor in urban systems. In croplands, rangelands, and wildlands land, over 50% of households fall below the relative poverty threshold. In rangelands, over 25% of households fall into the bottom consumption decile. While levels of poverty and extreme poverty tend to increase as the intensity of infrastructure decreases, consumption-based poverty levels are slightly lower in wildlands than rangelands.

On the upper end, over 87% of households in the top three deciles are in urban systems. Affluent households make up a larger percentage of the population in more developed systems. About 43% of the urban population are among the highest 30% of consumers, while 15% are in the highest 10%. However, using a national distribution to compare living standards would exclude many poor households in higher cost of living areas like Accra and Kumasi. Thus, in the following sections we use regional distributions to help adjust for differences in relative poverty across locations.

### 4.2. Consumption deciles and measures of well-being

**Figure 3.**
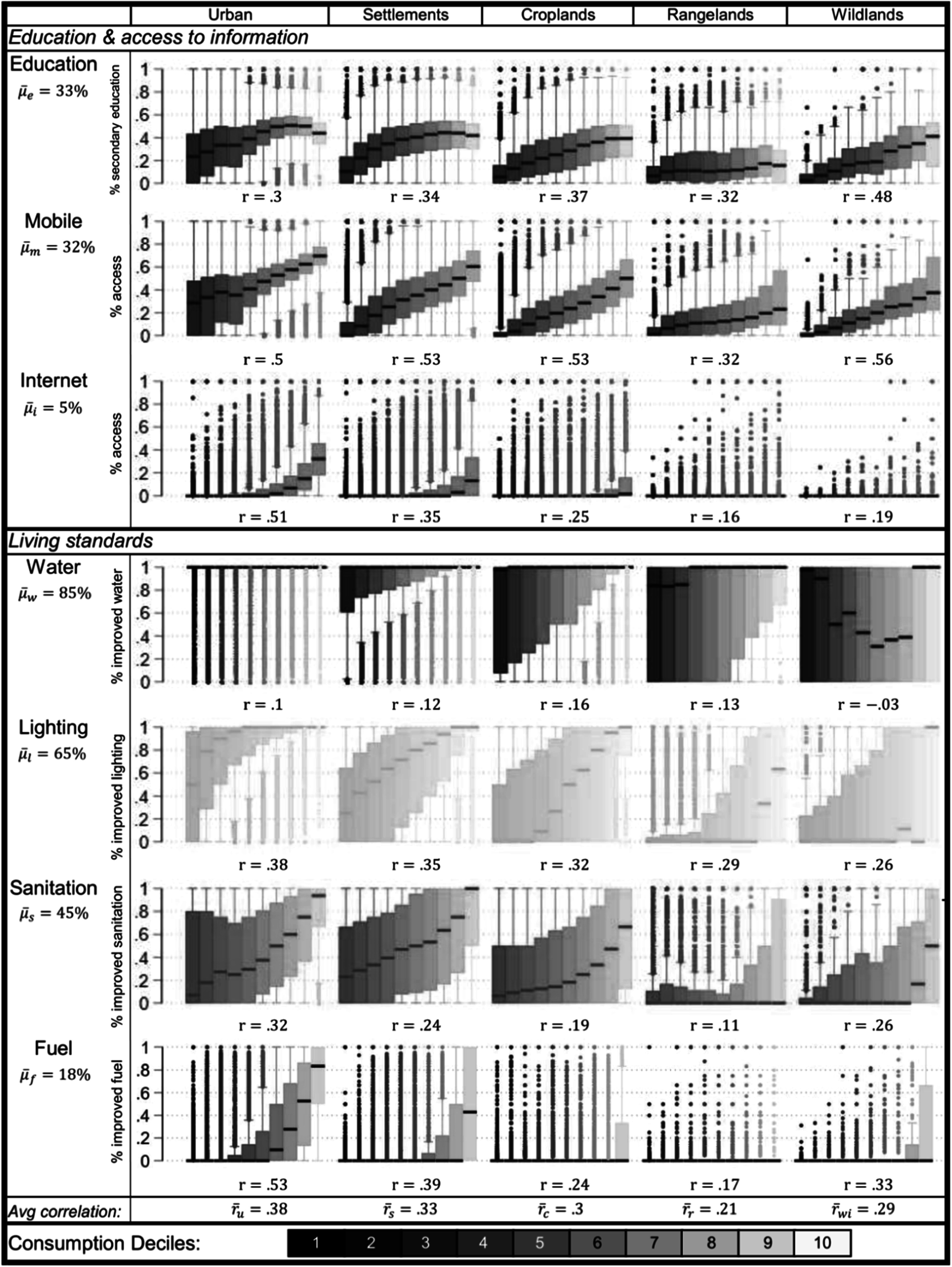
Measures of well-being. Box plots show the median and interquartile range of EA-level rates of improved well-being measures for households by consumption decile and SES type. Consumption deciles were defined for each region to reflect differences in relative poverty. Average rates of improved status (${\mathop{\mu }\limits^{\unicode{x00305}}}_{x}$) for each row describe the overall rate of improvement for that SES type. Percentages for each cell show the average improvement rate across consumption deciles for that SES type. Consumption deciles are represented from lowest to highest, represented by shaded and tinted hues, respectively.

Figure 3 shows the distribution of each well-being indicator by regionally defined deciles of consumption within SES types. We use regionally defined deciles to account for differences in poverty that may be felt differently across different regions. SES types are ordered from left to right by intensity of land use. Urban areas have the highest proportion of households with improved measures of well-being across all indicators (on average, 48.5%), especially in access to improved water and lighting (93% and 80%, respectively). This demonstrates a distinct material advantage of living in urban areas over the next most developed SES (settlements at 35%). Rates of improved well-being metrics are lowest in rangeland (on average 20%) and wildland (on average 20.9%) areas, showing very different general standards of living in these areas. These findings hold regardless of where the urban areas are located. Disaggregating urban areas based on the SES type of their surrounding district, shows that well-being is highest when the built environment is surrounded by urban districts and decreases in less developed districts. However, urban areas in more rural SES are better off than their rural counterparts, suggesting urban processes that affect well-being are common throughout the country (SI figure 1).

Across all well-being indicators, the proportion of households with improved metrics are positively correlated with consumption levels in all SES types. Well-being measures and consumption are significantly positively associated in most cases. The strongest correlations with consumption for all well-being metrics are in urban areas (${\mathop{r}\limits^{\unicode{x00305}}}_{u}$ = 0.38), with settlements and croplands showing similar patterns (${\mathop{r}\limits^{\unicode{x00305}}}_{s}$ = 0.33 and ${\mathop{r}\limits^{\unicode{x00305}}}_{c}$ = 0.30). Rangelands and wildland areas, however, show weaker associations across the board (${\mathop{r}\limits^{\unicode{x00305}}}_{r}$ = 0.19 and ${\mathop{r}\limits^{\unicode{x00305}}}_{w}$ = 0.29) and are notably different than the more market-integrated SES types (urban, settlement, and cropland areas) in improved outcomes for internet, sanitation, and fuel. For example, in rangelands, the correlation between consumption and sanitation is only 0.11, and only marginally stronger in wildlands at 0.26. The strongest associations in rangelands and wildlands are with rates of mobile phone ownership, as is the case across all SES types, but improved water sources are weakly *negatively* correlated with consumption in wildlands. Overall, rangelands, and in some cases wildlands, show weaker and more inconsistent relationships with living standard outcomes than the more market-integrated SES types.

### 4.3. Inequality in consumption and well-being measures across SES types

**Figure 4.**
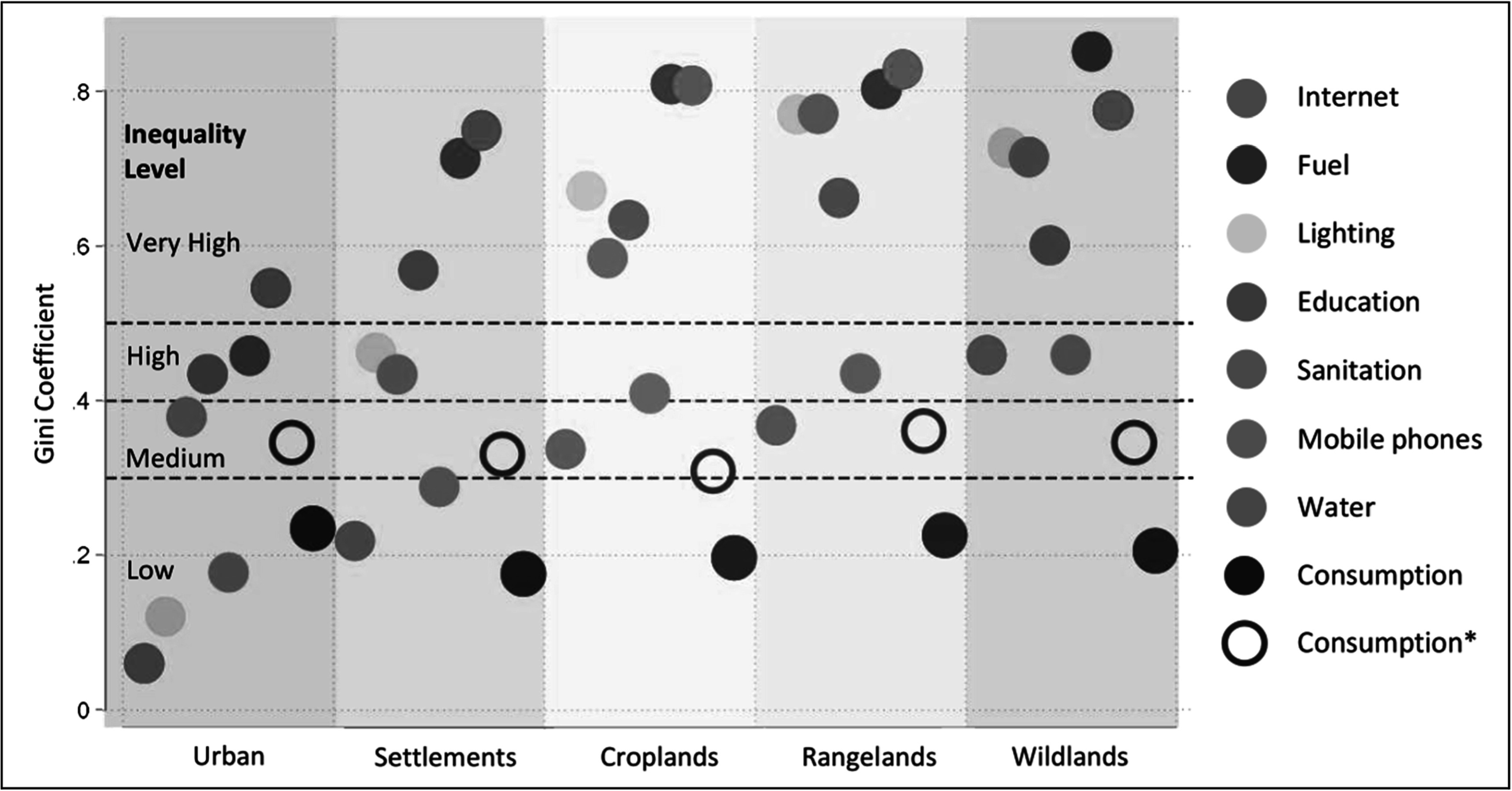
Inequality across SES types. Gini coefficients for consumption and well-being measures for each SES type. The color of the symbols corresponds to a well-being metric. Solid circles represent the Gini with respect to EA-level percent of households with improved metrics or average consumption. The hollow circle (*) represents the Gini calculated from estimates of household-level consumption.

Figure 4 shows Gini coefficients for consumption and well-being measures for each SES type. We have included two measures of consumption inequality: (1) EA-level consumption inequality, and (2) household-level consumption inequality denoted by *. EA-level consumption inequalities are low, around 0.20 for all SES types. Household consumption inequalities are higher (greater than 0.30), reaching medium levels of inequality. Regardless of measure, consumption inequalities are relatively similar across SES types. Other measures of inequality are not only typically much higher, but they are also far more varied between SES types. Only in urban and village systems are there inequalities lower than consumption (i.e. water and mobile in both, lighting in urban SES), indicating relatively equal access to these services and goods in more developed areas. Unlike consumption, inequality in most other measures of well-being qualifies as high or extremely high in less developed SES types. In settlements, five of eight metrics are high. In croplands and rangelands this is the case for six out of eight, and in wildlands seven out of eight measures. Typically, fuel, internet, lighting, and sanitation inequality are the highest, followed by mobiles, water, and consumption inequality. In wildland areas, only consumption inequality is considered low. Ultimately, places that are more ecologically dependent and less developed, have the highest levels of inequality for almost all well-being measures.

## 5. Discussion

In developing evidence from microdata linked to the enumeration area-level, we are able to explore inequalities in Ghana with a spatial lens. Not only do we demonstrate that levels of well-being differ between SES contexts, but the spatial granularity of our data also allows us to show that places within a particular SES experience vast differences in material well-being. We first discuss our findings as they relate to well-being across SES types, then inequalities within SES, and finally overall development policy. We make suggestions on how to tailor policy to different SES contexts.

### 5.1. Well-being across SES

Our first general finding is that our measures of well-being are, for most of the population, positively related to consumption. In the three most developed systems (urban, settlements, and croplands), there is a strong association between consumption and improved well-being. The association with consumption is notably weaker or inconsistent in rangelands and wildlands.

Education and living standards measures have similar relationships with consumption in urban areas, settlements, and cropland systems. Livelihoods in these three SES types are largely market-oriented, albeit with different types of economic activity, levels of infrastructure, and market integration. Rangelands and wildlands, in contrast, have smaller populations and environmental and economic activities are often less market-oriented. While some rangeland production is commercial, much is for local or individual consumption, especially in northern Ghana [79, 80]. Rangelands have the lowest average level of consumption of any SES type, with particularly low levels for some well-being metrics, even among the highest consumption deciles. Physical access to goods and services may be limited in pastoral-based systems, or preferences are simply different. As is well documented, rangeland and pastoral systems often prioritize different sets of material goods and patterns of consumption, and store wealth in ‘productive assets’ (i.e., livestock) [19, 81]. Our data support the idea that to understand well-being in rangelands, one must consider metrics beyond consumption and standard measures of well-being.

Wildland systems are the least populous and mostly forested. The northern-most district is part of the Guinean savannah, which is dominated by short trees, grass, and scrubland. The two central wildland regions are located in the mixed transitional zone, while the southern-most district is characterized by wet evergreen forest. Large portions of these districts are state protected national parks (i.e. Mole, Digya, and Bui National Parks & Ankasa Conservation Area). Local communities are often highly dependent on the local environment, and access to resources within the parks is limited, and thus can have significant impacts on local livelihoods [82]. Wildland SES have the lowest levels of infrastructure development, but do not always have the lowest levels of well-being.

In summary, consumption is a fair proxy for well-being in more developed SES types. However, in SES types that are not as integrated into the market economy and more ecologically embedded, different internal logics govern strategies to ensure quality of life and meet one’s basic needs such that consumption is a weaker indicator of well-being.

### 5.2. Inequalities across SES

Our data show that consumption inequality is a poor predictor of inequality across a broad range of well-being metrics. Consumption inequality is low, and varies little, across all SES types, in contrast to inequalities in other well-being metrics that increase with decreasing intensity of development. In fact, consumption inequality is highest in the urban SES, the opposite of all other well-being measures, where above we note that consumption tracks most closely to well-being.

Thus, we see there are wide differences in inequality across our well-being metrics. Consumption and income are justified as a global proxy for well-being based on the idea that fungible wealth can be traded for goods or materials that improve quality of life [83]. Yet we see here that while consumption may be a broad indicator of well-being in most systems, it does not provide a good measure of the material inequalities that can exist, especially for SES that are more ecologically embedded.

High inequalities in living standards, but low inequalities in consumption, could also relate to higher consumption of goods or materials that were not measured in the standard set of goods in the census. This may be the case, especially in rangeland and wildland systems where livelihood strategies and thus consumption patterns can be quite different compared to the more market-integrated urban, settlement, and cropland systems.

With respect to both poverty and inequality metrics, future work could extend this analysis to include comparable data from the 2021 Census (when spatially resolved micro data become available). This will allow for a more dynamic picture of how poverty and inequality have changed over time, and how patterns may relate to different social-ecological processes.

### 5.3. Policy implications

Our findings highlight how development and poverty alleviation programs should recognize both social and ecological contexts to better incorporate the challenges unique to local environments. For instance, in urban areas, where there are large numbers of poor but better conditions than elsewhere, it may be preferable to focus on improving specific material dimensions where the poor lag behind (e.g., fuel, education, ICT). However, basing decisions on a rural-urban divide would be misleading. Our evidence points more closely to differences between what we see as more market-oriented (urban, settlement, cropland) versus more environmentally dependent (rangeland, wildland) SES types. In more market-oriented rural SES of settlements and cropland areas, patterns in well-being and inequality are more similar to urban areas than rangeland and wildland rural SES. Market-oriented areas may be better served by focusing on improving conditions for the poor while improving market access in these areas. In more ecologically oriented SES types, where more households struggle to meet basic needs, development efforts could aim to improve basic services in a locally- and contextually-relevant ways. For example, policies that work in settled and agricultural communities often do not reflect the ecological or social needs of pastoral communities [84–86]. Those inhabiting ‘wildlands’ are also supported by a range of livelihoods—from indigenous forest-dwelling communities, to tourism, to park and wildlife management—that may necessitate different policy approaches to help support local communities and reduce inequality [87–89]. Many of these challenges involve complex issues around land rights [90], political and power dynamics [91, 92], and private versus public benefits from land [93, 94]. Co-production of viable policies and continuous engagement with local communities is necessary for understand development needs, wants, and modes of delivery [95–97].

While co-produced solutions will likely lead to the best outcomes, we have some ideas about policy interventions that might work in various areas [96]. For example, unconditional cash transfer programs can improve health and educational outcomes [98] but are likely more impactful for households in market integrated systems (urban, settlements, croplands) than rangelands and wildland residents. Considering place and SES when designing programs to improve access to infrastructure and services, could help policy have an impact beyond the poorest and raise well-being for the whole population.

In SES such as croplands, rangelands, and wildlands, where households are more dependent on farming, pastoral, or subsistence livelihoods, assisting in developing resilient local food systems may be relevant [99]. Land-dependent communities are more susceptible to the impacts of climate change, which places pastoralists and farmers at risk of deeper deprivation, making it important to secure household livelihoods and improve food security. Co-producing possible policy mechanisms with communities will best lead to robust solutions. One flexible initiative in Ghana is the Ghana School Feeding Programme, implemented at the school-level, which has a long-term goal of reducing poverty and malnutrition through strengthening local food production and consumption systems [100]. Sourcing foods that are locally produced for school meal programming not only improves nutrition for school aged children, but also provides a local market for small producers.

Households with limited financial resources must optimize livelihoods and their own well-being, often constrained by the local ecological or available environmental resources, services, and infrastructure. A standardized set of living standard measures may not appropriately capture these conditions, nor the preferences or cultural norms of local populations particularly in rangelands and wildland contexts. These considerations could be addressed by working with local communities to understand what they view as quality of life/well-being metrics through co-produced or participatory poverty and wealth assessments. Recent literature provides guidance on developing local indicators of well-being [101–103], including the Basic Necessities Survey [104]. These provide promise in developing locally produced reflections of community needs, but further work is needed to see how well these measures might empirically correlate with consumption or income. A paradox seems to exist in developing indicators that are simultaneously locally salient and relevant but also comparable over time and across communities.

## 6. Conclusion

Socio-ecological systems shape the experience of poverty and inequality. This adds nuance to our understanding of inequality between (and within) urban and rural contexts, and allows us to identify the challenges that households face in different socio-ecological contexts across Ghana. Here, we find that SES types that are more highly integrated into the market economy have higher rates of consumption (in monetary terms) and lower levels of material inequality. Unsurprisingly, more rural and less developed socio-ecological systems face the deepest deprivation along more dimensions. We find, on the one hand, that consumption is a relatively good proxy for levels of well-being, however, it is only weakly associated in less developed contexts. On the other hand, monetary-based inequality is a poor predictor of well-being inequality. Thus, it is appropriate to consider how context might shape well-being and evaluate how well measures of economic capacity, like consumption, relate to material and living standards, and inequality, in interrogating and designing policies to reduce deprivation. Using microdata from Ghana to evaluate multiple dimensions of deprivation allows us to see that in the least developed contexts there are many indicators where poor and even middle-income households are deprived, and only a small number of households have access to improved conditions. In urban areas, the poor are deprived among particular dimensions, while higher consumption groups are better able to meet their basic needs. Our approach provides evidence on *where* particular concerns overshadow others, *which* issues are most acute, and *who* lacks access to basic services in Ghana. Applying this framework to other ecologically diverse contexts can help policy makers understand the most pressing challenges and how to develop programs with communities to help meet their needs.

## Data Availability

The data that support the findings of this study are openly available at the following URL/DOI: https://doi.org/10.5281/zenodo.10669077.
